# Hard and Soft Water

**Published:** 1888-03-24

**Authors:** 


HARD AND SOFT WATER.

Hard water, so-called, contains an excess or large quantity of salts of lime and magnesia. Those, on the other hand, from which those salts are absent or nearly so, are termed soft waters. Amongst hard waters may be claimed those supplied by the metropolitan water companies, in which are present about twenty grains per gallon of salts of lime and magnesia. Examples of soft water are afforded by that of Loch Katrine, in Scotland, containing in the gallon a scarcely appreciable amount of these ingredients, and that of Bala Lake, in North Wales, in which there is less than a quarter of a grain of lime and magnesia salts per gallon. Carbonate of lime held in solution by carbonic acid is, for the most part, the hardening ingredient in the waters of the London companies. These waters can be rendered softer by boiling, which process, driving off the carbonic acid, converts the previously soluble lime salt into an insoluble one, which falls to the bottom of the vessel and may be removed from the water. The deposit on kettles and boilers which has been formed in this manner is chiefly insoluble carbonate of lime.

